# Polyphenols as Modulators of Gastrointestinal Motility: Mechanistic Insights from Multi-Model Studies

**DOI:** 10.3390/ph18101564

**Published:** 2025-10-16

**Authors:** Andrzej Chomentowski, Krzysztof Drygalski, Tomasz Kleszczewski, Marta Berczyńska, Marzena Tylicka, Jacek Kapała, Agnieszka Raciborska, Przemysław Zubrzycki, Hady Razak Hady, Beata Modzelewska

**Affiliations:** 1 Department of Biophysics, Faculty of Medicine, Medical University of Bialystok, 15-222 Bialystok, Poland; chomentowskiandrzej@gmail.com (A.C.); tomasz.kleszczewski@umb.edu.pl (T.K.); berczynskam@gmail.com (M.B.); marzena.tylicka@umb.edu.pl (M.T.); jacek.kapala@umb.edu.pl (J.K.); agnieszka.raciborska@umb.edu.pl (A.R.); 2 Division of Hypertension & Diabetology, Faculty of Medicine, Medical University of Gdansk, 80-307 Gdansk, Poland; drygalskikrzysztof@gmail.com; 3 2nd Clinical Department of General, Gastroenterological and Oncological Surgery, Medical University of Bialystok, 15-222 Bialystok, Poland; przemek.zubrzycki1997@gmail.com (P.Z.); razak.hady@umb.edu.pl (H.R.H.)

**Keywords:** gastrointestinal smooth muscle, intestinal motility, ion channel modulation, muscarinic receptors, polyphenol bioavailability, pharmacokinetics, drug–nutrient interactions, herbal extracts, translational pharmacology

## Abstract

Dietary polyphenols are recognized as crucial modulators of gastrointestinal motility, holding therapeutic promise for conditions like irritable bowel syndrome, postoperative ileus, and functional dyspepsia. However, their reported effects are heterogeneous, ranging from spasmolytic to prokinetic. This review aims to clarify these inconsistencies by synthesizing experimental evidence on structure–activity relationships and underlying mechanisms. Relevant publications were identified in PubMed and Google Scholar using terms related to polyphenols and gastrointestinal motility. References were selected for relevance, and the narrative review integrates findings from in vitro, ex vivo, in vivo, and clinical studies. Across various experimental models, polyphenols function as multi-target modulators of gastrointestinal smooth muscle. The primary mechanisms identified involve the blockade of voltage-dependent L-type Ca^2+^ channels, activation of K^+^ channels (BK, K_ATP_), and modulation of the NO/cGMP and cAMP/PKA pathways. Flavones and multiple flavonols consistently demonstrate spasmolytic activity via Ca^2+^ channel antagonism. In contrast, flavanones engage BK and K_ATP_ channels to induce membrane hyperpolarization. Complex extracts from plants like ginger and turmeric exhibit mixed pro- or antimotility effects, reflecting the diverse profiles of their constituent compounds. While robust ex vivo pharmacology and some in vivo and human data exist, a high degree of dataset heterogeneity and inconsistent reporting impedes direct translational efforts. Polyphenols are promising multi-mechanistic modulators of gastrointestinal motility with clear structure–activity patterns. To advance their clinical application, future research must focus on establishing standardized in vivo pharmacokinetics, conducting targeted structure–activity studies, employing bioassay-guided fractionation, and designing rigorous clinical trials.

## 1. Introduction

Gastrointestinal (GI) motility, orchestrated by the coordinated activity of interstitial Cajal cells, enteric neurons, and GI smooth muscle cells, plays a fundamental role in processes such as digestion, nutrient absorption, and waste elimination. Dysregulation of this complex system can lead to a diverse array of clinical manifestations, including functional GI disorders like irritable bowel syndrome (IBS), functional dyspepsia, or postoperative ileus [1]. However, existing pharmacological treatments for motility disorders frequently exhibit limited effectiveness and are often associated with adverse effects, underscoring the critical imperative for the development of novel, more precise, and safer therapeutic strategies [2,3].

Polyphenols, a chemically diverse class of secondary plant metabolites, have attracted significant attention owing to their wide-ranging biological properties, including antioxidant, anti-inflammatory, and neuromodulatory effects [4,5,6]. Within this class, flavonoids represent the most prevalent and structurally versatile subclass, commonly found in fruits, vegetables, herbs, and various traditional medicinal preparations. Growing evidence indicates that polyphenols may influence GI motility by modulating key elements such as ion channels, neurotransmitter systems, and smooth muscle contraction mechanisms.

Despite a continuously expanding interest in the therapeutic potential of polyphenols for GI health, their precise effects on gut motility remain incompletely characterized. While certain polyphenols exhibit clear spasmolytic properties, others might exert prokinetic effects, depending on specific chemical structure, applied concentration, and the prevailing physiological or pathological condition of the GI tract.

This review offers a narrative, mechanistically oriented overview of the contemporary experimental evidence concerning the effects of polyphenols (with a specific focus on flavonoids) on GI smooth muscle contractility. This organization, categorized by flavonoid subclass, aims to elucidate structure-activity relationships, delineate common mechanistic pathways, and identify lead candidates with potential for subsequent clinical translation.

## 2. Methods

This review was prepared as a narrative synthesis of the literature to integrate current knowledge on the effects of polyphenols on GI motility. To gather relevant material, we consulted electronic databases such as PubMed and Google Scholar. Search terms included “polyphenols”, “flavonoids”, “gastrointestinal motility”, “smooth muscle”, and “contractility”, alone or in combination.

Priority was given to original research articles reporting mechanistic insights from in vitro, ex vivo, in vivo, or clinical studies, as well as to authoritative reviews that provided broader context. References were selected based on their relevance to the topic rather than through predefined systematic inclusion and exclusion criteria.

The review is organized by polyphenol subclasses, followed by mechanistic pathways and evidence from both isolated compounds and complex extracts. This structured presentation was chosen to improve clarity and highlight structure–activity relationships. However, the approach remains narrative in nature, aiming to provide a comprehensive and mechanistically oriented overview rather than an exhaustive or systematic synthesis of the literature.

## 3. Classification and Chemistry of Polyphenols

**Figure 1:**
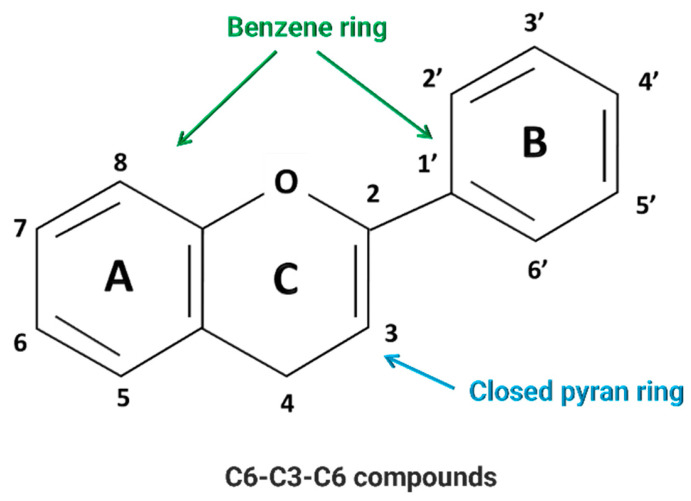
The basic skeleton structure of flavonoids.

**Figure 2:**
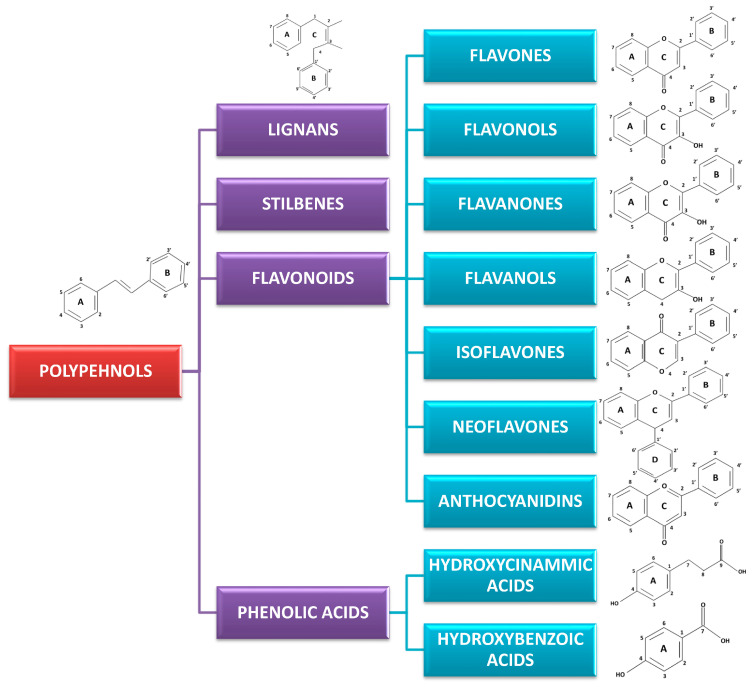
Classification of polyphenols.

Polyphenols are a broad class of plant-derived compounds characterized by a chemical structure containing one or more hydroxyl groups directly bonded to at least one aromatic (phenyl) ring. Structurally, they are primarily categorized into two major groups: flavonoids, defined by their characteristic C6-C3-C6 carbon skeleton, and non-flavonoids (Figure 1 and Figure 2) [7,8].

Among polyphenols, flavonoids represent a highly prevalent and structurally diverse class, widely distributed across various dietary sources, including fruits, vegetables, herbs, and beverages such as tea and wine. Flavonoids are derivatives of 2-phenyl-benzo-γ-pyrone (2-phenyl-3,4-dihydro-2*H*-1-benzopyran-4-one). Their defining structural characteristic is a common diphenylpropane backbone, consisting of two aromatic rings (A and B) linked by a linear three-carbon chain (Figure 1). The three-carbon (-C3-) may be included through an oxygen bond between the two phenyl rings into a five-membered heterocyclic ring (furan) as in aurones or a six-membered heterocyclic ring (pyran) to give flavonoids, which constitute the largest group. Flavonoids occur as aglycones, glycosides, and methylated derivatives. Further classification of flavonoids into six primary subclasses, namely flavones, flavonols, flavanones, flavanols (catechins), isoflavones, neoflavones, and anthocyanidins, is determined by the oxidation state and substitution pattern of this C-ring (Figure 2).

Flavones, exemplified by compounds such as apigenin and luteolin, are structurally defined by a C2-C3 double bond and a C4 ketone group within their heterocyclic C-ring. Commonly, they feature hydroxylation at positions 5 and 7 of the A-ring and position 4 of the B-ring (Figure 1) [9,10].

Flavonols, including well-known examples such as quercetin, kaempferol, and myricetin, are distinguished from flavones by the presence of a hydroxyl group at the C3 position of their heterocyclic C-ring (Figure 1). This additional -OH not only enhances their antioxidant capacities, but also significantly influences their interactions with various biological targets, including ion channels and enzymes [11,12,13].

Flavanones such as naringenin and hesperidin are characterized by the absence of a C2=C3 double bond in their C-ring, which typically confers greater structural flexibility. These compounds are highly prevalent in citrus fruits and frequently occur in their glycosylated forms [14,15].

Flavanols, also known as catechins, with representatives including catechin, epicatechin, are distinct saturated flavonoids lacking both the C2=C3 double bond and the 4-keto group. These compounds are particularly abundant in tea, cocoa, and various fruits. Their galloylated derivatives often exhibit enhanced bioactivity [16,17].

Isoflavones, with representative compounds such as genistein and daidzein, are uniquely characterized by the attachment of their B-ring at the C3 position of the C-ring and are primarily found in soy products. Their structural similarity to estrogen allows for them to act as phytoestrogens. Still, their effects on GI smooth muscle are distinct and varied, often involving direct modulation of ion channels and second messenger systems [18,19].

Neoflavones, with examples like dalbergin and coutareagenin, are a minor subclass of flavonoids characterised by attachment of the B-ring at the C4 position of the C-ring. They have diverse biological activities with recent research suggesting their chemoprotective capabilities [20,21].

Anthocyanidins, including examples like malvidin and petunidin, are positively charged chromophores responsible for imparting red, purple, and blue hues to numerous fruits and flowers. Given their inherent structural instability at neutral pH, they are predominantly found in nature as their more stable glycosylated forms, known as anthocyanins [22]. Although known for their potent antioxidant properties, their direct effects on GI smooth muscle are less well-defined.

Post-synthetic modifications, including hydroxylation, methylation, glycosylation, and acylation of these core flavonoid structures, substantially augment their chemical diversity. These modifications critically impact their bioavailability, metabolic fate, and overall biological activity. For example, the conjugated C2=C3 double bond and 4-keto group present in flavones and flavonols are typically linked to more potent myorelaxant effects in gastrointestinal smooth muscle, a phenomenon potentially mediated by enhanced interaction with L-type calcium channels [8].

Beyond the expansive class of flavonoids, several other major polyphenol categories (non-flavonoids), notably phenolic acids, stilbenes, and lignans, also hold significant relevance for GI function.

Phenolic acids are classified into hydroxybenzoic acids and hydroxycinnamic acids. They comprise derivatives of both benzoic acid (gallic acid) and cinnamic acid (caffeic acid, ferulic acid, rosmarinic acid). Structurally, they are characterized by a single aromatic ring featuring various hydroxyl and/or methoxy substitutions, often extended by carboxylic acid or other polar functional groups [23]. These compounds are ubiquitous in plant-derived foods, with high concentrations particularly noted in herbs, coffee, and whole grains. Their comparatively small molecular size contributes to efficient absorption in the upper GI tract, and many demonstrate potent local anti-inflammatory and antioxidant activities [24].

Stilbenes, such as resveratrol and pterostilbene, are characterized by a C6-C2-C6 carbon skeleton, comprising two aromatic rings connected by an ethylene bridge (Figure 1). Although less ubiquitously distributed in nature compared to other polyphenol classes, stilbenes are present in notable quantities in grapes, berries, and peanuts. They are recognized for their potent antioxidant and anti-inflammatory attributes [25,26].

Lignans are structurally formed via the oxidative dimerization of two phenylpropanoid (C6-C3) units (Figure 1). These compounds are primarily encountered in seeds, whole grains, and certain vegetables. Key representative compounds include secoisolariciresinol and matairesinol. Crucially, within the human gut, lignans undergo biotransformation by the resident microbiota into enterolignans, such as enterodiol and enterolactone, which possess mild estrogenic and anti-inflammatory activities [8].

## 4. Mechanisms of Action of Polyphenols on Gastrointestinal Smooth Muscle

**Figure 3:**
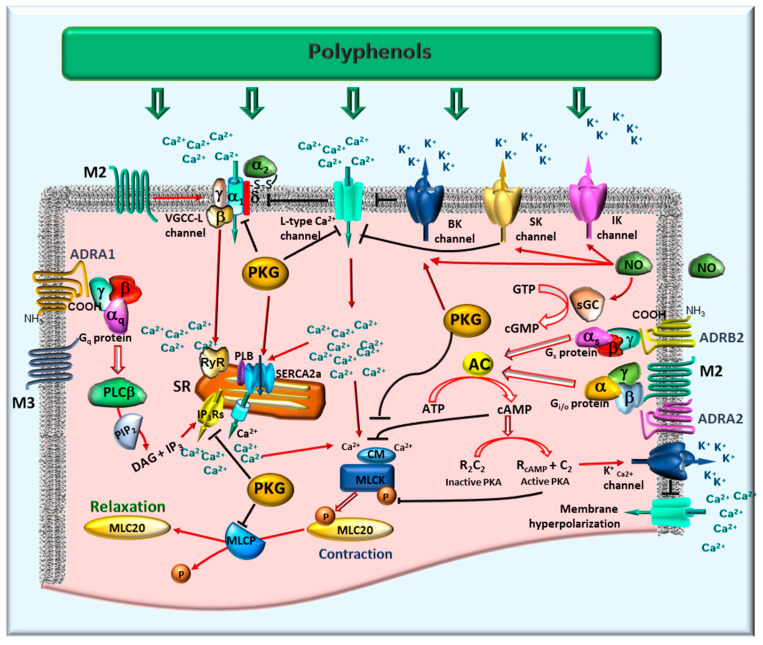
Proposed mechanisms of polyphenol action on gastrointestinal (GI) smooth muscle contractility. Polyphenols primarily modulate calcium (Ca^2+^) levels, both by inhibiting voltage-gated calcium channels (VGCCs) to reduce extracellular Ca^2+^ influx [27] and by regulating Ca^2+^ release from the sarcoplasmic reticulum (SR) via modulation of inositol trisphosphate receptors (IP_3_Rs) and ryanodine receptors (RyRs). They also engage with key receptor systems, notably the muscarinic M3 (M3) and alpha-1 adrenergic (ADRB1) receptors, which are typically coupled to Gq-proteins. This interaction activates phospholipase C beta (PLCβ), initiating the hydrolysis of phosphatidylinositol 4,5-bisphosphate (PIP_2_) to generate diacylglycerol (DAG) and inositol trisphosphate (IP_3_). IP_3_ subsequently stimulates IP_3_Rs on the SR, promoting Ca^2+^ release and triggering contraction. Simultaneously, polyphenols modulate signaling through muscarinic M2 (M2) receptors (G_i/o_-coupled) and beta-2 adrenergic (ADRB2) receptors (G_s_-coupled proteins [28]. These interactions influence adenylate cyclase (AC) activity, resulting in altered cyclic adenosine monophosphate (cAMP) levels. Elevated cAMP, alongside cyclic guanosine monophosphate (cGMP) (often stimulated by nitric oxide, NO, as described below), activates protein kinase A (PKA) and protein kinase G (PKG), respectively. PKA and PKG facilitate smooth muscle relaxation by stimulating myosin light-chain phosphatase (MLCP). MLCP dephosphorylates myosin light chain 20 (MLC20), thereby reducing the contractile force. Conversely, smooth muscle contraction is predominantly initiated by MLC20 phosphorylation, catalyzed by myosin light-chain kinase (MLCK) [27]. Additionally, polyphenols influence NO production, which activates soluble guanylate cyclase (sGC), thereby enhancing cGMP-mediated signaling and ultimately promoting relaxation [29]. Direct modulation of potassium channels (including large-conductance calcium-activated potassium (BK), small-conductance calcium-activated potassium (SK), and intermediate-conductance calcium-activated potassium (IK) channels) further contributes to membrane potential regulation and thus contractility [30,31]. Lastly, polyphenols can impact Ca^2+^ reuptake into the SR through sarco/endoplasmic reticulum Ca^2+^-ATPase 2a (SERCA2a), an enzyme regulated by phospholamban (PLB), thereby regulating intracellular Ca^2+^ dynamics [32]. Arrows denote stimulatory effects; black lines represent inhibitory effects.

Smooth muscle is a fundamental contractile tissue governing critical functions across organ systems, including the regulation of vascular tone, airway caliber, and pupil diameter. Within the GI tract, its precisely coordinated activity drives the motility patterns essential for the digestion and subsequent absorption of nutrients. Smooth muscle contraction and relaxation in the GI tract are precisely regulated processes that depend on the interplay of electrical excitability, intracellular calcium dynamics, contractile protein activation, and extensive neuromodulation by the enteric nervous system. Dietary polyphenols actively modulate these diverse regulatory levels, thereby influencing GI motility in both physiological states and pathological contexts (Figure 3).

### 4.1. Excitation-Contraction Coupling and the Role of Calcium

Smooth muscle contraction is fundamentally initiated by membrane depolarization, which leads to the opening of sarcolemmal voltage-gated L-type calcium channels (VGCCs). The subsequent influx of extracellular calcium elevates cytosolic calcium concentrations, prompting the formation of a calcium-calmodulin complex. This complex, in turn, activates myosin light chain kinase (MLCK), which phosphorylates the 20 kDa regulatory light chains (MLC20) of myosin II. This phosphorylation facilitates actin-myosin cross-bridge formation, thereby generating contractile force [33].

In GI smooth muscle, calcium influx can also be augmented by activation of ligand-gated channels or via store-operated calcium entry mechanisms. The force and temporal characteristics of contraction are determined by both calcium availability and the dynamic equilibrium between MLCK and myosin light chain phosphatase (MLCP) activities [32].

### 4.2. Relaxation Via Potassium Channels and Membrane Hyperpolarization

Smooth muscle relaxation typically ensues from repolarization or hyperpolarization of the membrane potential, thereby diminishing calcium influx through VGCCs. This phenomenon is principally mediated by the activation of various potassium channels, notably including the following:

- Large-conductance calcium-activated potassium (BK) channels: Sensitive to both membrane depolarization and elevations in intracellular calcium.
- ATP-sensitive potassium (K_ATP_) channels: Functionally link cellular metabolic status to membrane potential.
- Voltage-gated and inward rectifier potassium channels: Contribute significantly to the maintenance of basal tone and electrical excitability.

The efflux of K^+^ ions resulting from the opening of these channels drives the membrane potential toward more hyperpolarized states, consequently limiting excitatory calcium currents [30,31].

### 4.3. Regulation by Nitric Oxide and the cGMP Pathway

Nitric oxide (NO) exerts a fundamental inhibitory role in GI motility, primarily via its action on non-adrenergic, non-cholinergic inhibitory neurons within the enteric nervous system. In smooth muscle cells, NO activates soluble guanylate cyclase (sGC), leading to elevated intracellular cyclic guanosine monophosphate (cGMP) levels. cGMP subsequently activates protein kinase G (PKG), which in turn reduces intracellular calcium concentrations and inhibits the phosphorylation of contractile proteins, thereby promoting relaxation. Furthermore, evidence suggests that NO directly modulates specific potassium channels, including BK, small-conductance (SK), and intermediate-conductance (IK) calcium-activated potassium channels. These direct effects also contribute to membrane potential regulation and, consequently, to GI tract contractility [34,35].

This pathway is crucial for processes such as descending relaxation during peristalsis, modulation of lower esophageal sphincter tone, and adaptive gastric relaxation. Its regulation can occur at multiple levels, encompassing NO synthesis by nitric oxide synthase (NOS), sGC activation, and cGMP degradation by phosphodiesterases [29].

### 4.4. Modulation of Contractile Machinery: MLCK and MLCP

Independent of direct calcium signaling, the magnitude of contraction is regulated by the balanced activities of MLCK and MLCP. While MLCK drives contraction through MLC20 phosphorylation, MLCP counteracts this process by dephosphorylating MLC20, thereby promoting relaxation. Notably, MLCP activity is itself subject to regulation by Rho-associated protein kinase (ROCK) and protein kinase C (PKC). These kinases can inhibit MLCP, consequently prolonging contraction and contributing to calcium sensitization.

Therefore, compounds that inhibit MLCK, activate MLCP, or interfere with the RhoA/ROCK signaling pathway can effectively reduce contractility, even in situations where intracellular calcium levels are elevated [32,33].

### 4.5. Neurotransmitter Receptor-Mediated Modulation

GI smooth muscle function is extensively modulated by the enteric nervous system, which integrates diverse inputs from various neurotransmitters and hormones. Key receptors mediating motility regulation include the following:

- Serotonin receptors: These receptors critically modulate smooth muscle contraction and relaxation. The precise balance between activating excitatory subtypes (e.g., 5-HT_3_, 5-HT_4_, 5-HT_2_) and inhibitory subtypes (e.g., 5-HT_7_) enables motility adapted to specific digestive requirements [36,37].
- Adrenergic receptors (ADRA): These receptors can elicit either relaxation or contraction depending on their specific distribution and subtype. ADRA1 receptors typically promote contraction and sphincter tone. In contrast, ADRA2 receptors inhibit neurotransmitter release, thereby diminishing contraction, while ADRB2 receptors mediate smooth muscle relaxation. Collectively, these subtypes regulate gut motility and tone in response to sympathetic innervation [28].
- Muscarinic acetylcholine receptors (mAChRs): The M2 and M3 subtypes are predominantly involved in intestinal smooth muscle. M3 receptors serve as the principal mediators of smooth muscle contraction. Their activation, coupled to G_q/11_ proteins, stimulates phosphoinositide hydrolysis, leading to the production of inositol trisphosphate (IP_3_). IP_3_ then triggers Ca^2+^ release from the sarcoplasmic reticulum (SR), elevating intracellular Ca^2+^ concentration. This Ca^2+^ increase activates calmodulin and, consequently, MLCK, thereby promoting MLC20 phosphorylation and initiating contraction [36]. M2 receptors constitute approximately 80% of muscarinic receptors in GI smooth muscle. Coupled to G_i/o_ proteins, they primarily inhibit adenylate cyclase (AC), resulting in reduced cyclic AMP levels. Although M2 receptors do not directly induce contraction, they enhance the contractile response by increasing the Ca^2+^ sensitivity of the contractile apparatus and modulating ion channel activity, specifically by inhibiting K^+^ currents and modulating VGCCs. The synergistic activation of M2 and M3 receptors leads to membrane depolarization via the generation of non-selective cationic and chloride currents, further facilitating Ca^2+^ influx and contraction [36]. Significantly, the contractile contribution of M2 receptors is conditional on M3 receptor activation; antagonism of M3 receptors typically abolishes the contractile response, indicating that M3 receptors are indispensable for initiating contraction, whereas M2 receptors serve to modulate and sustain it [37].
- Upper GI smooth muscle of porcine expresses both M2 and M3, with variable ratios in different GI regions [38]. The coexistence of M2 and M3 receptors in the intestine ensures a balanced cholinergic control [39]. Immunohistochemical studies and mRNA expression analysis in colonic samples confirmed the presence of M1, M2, and M3 receptors, with M2 and M3 immunoreactivity particularly abundant in circular and longitudinal muscle layers, as well as within elements of the enteric nervous system [40]. The functional significance of M2 receptors in the GI tract has been supported by pharmacological studies and animal models, which demonstrate that M2, together with M3, contributes to cholinergic control of intestinal smooth muscle contraction and serves as a presynaptic autoreceptor modulating acetylcholine release [36].

However, these receptors are widely expressed in other tissues, raising the potential for off-target effects when modulated by polyphenols or extracts. M2 receptors are the dominant muscarinic subtype in the heart, where they mediate vagally induced bradycardia [27]. M3 receptors play crucial physiological roles across multiple organ systems. They mediate secretion in exocrine glands, particularly salivary and airway submucosal glands [41]. In the respiratory system, M3 receptors are the predominant subtype on airway smooth muscle, responsible for ACh-induced bronchoconstriction and airway hyperresponsiveness [42]. Within the cardiovascular system, M3 receptors located on vascular endothelial cells contribute to ACh-induced, endothelium-dependent vasodilation [43].

The involvement of both M2 and M3 receptors in GI motility underscores the complexity of cholinergic regulation. Polyphenols that interact with these receptors may therefore influence not only intestinal function, but also cardiovascular, respiratory, and glandular physiology, depending on their specificity and systemic exposure.

- Opioid receptors (μ, δ, κ) play an important role in modulating smooth muscle contraction and GI motility, primarily leading to the inhibition of both motility and secretion [44].

Compounds capable of modulating these complex neurotransmitter receptor systems can indirectly influence smooth muscle tone by altering neuronal input, rather than directly acting on the muscle cells themselves [45].

### 4.6. Inflammatory and Oxidative Stress Modulation

Under various pathophysiological conditions, including postoperative ileus, irritable bowel syndrome, or inflammatory bowel disease, GI contractile function can become compromised. This impairment often stems from cytokine-induced disruption of signaling pathways, oxidative damage, and underlying neuromuscular dysfunction. In this context, polyphenols capable of attenuating the expression of inflammatory mediators (e.g., iNOS, COX-2) or scavenging reactive oxygen species can play a crucial role in preserving or restoring contractile responsiveness. Thus, their anti-inflammatory and antioxidant activities represent significant indirect mechanisms of action [46].

## 5. Effects by Flavonoid Subclass: Unraveling Structure-Activity Relationships in GI Motility Modulation

This section explores the specific effects of various flavonoid subclasses on GI smooth muscle contractility. By examining individual compounds and their mechanisms, we aim to delineate the structure-activity relationships that govern their influence on GI motility, providing crucial insights for potential therapeutic applications (Table 1).

Experimental reports differ in whether they test chemically defined single polyphenols or complex botanical extracts. In Section 5, Section 6, Section 7 and Section 8, we prioritize studies that explicitly identify the active molecule(s). When only extracted data are available, we clearly indicate this limitation. To facilitate quantitative comparison, Table 1 and Table 2 list EC_50_ values or concentration ranges for individual compounds or extracts whenever these were reported. This approach highlights mechanistic evidence while acknowledging the limitations inherent to extract-based studies (variable composition, matrix effects, and potential synergy) [7,47,48].

**Table 1:** Effects of pure polyphenolic compounds on gastrointestinal smooth muscle contractility.

Compound	Tissue/Model	Main Effect/Mechanism	EC_50_ and/or Reported Concentration	Reference
**Luteolin**	Mouse colon/Murine model (ex vivo)	Relaxation/L-type Ca^2+^ channel antagonism	10–30 µM	[10]
**Apigenin**	Mouse stomach; guinea pig ileum (ex vivo)	Relaxation/Ca^2+^ channel antagonism	not specified	[49]
			12.5 µM	[50]
			1.02 µM/0.1–100 µM	[9]
**Naringenin**	Rat colon (ex vivo); Rat uterus (in vivo); Guinea pig intestinal (ex vivo)	Relaxation/BK activation, hyperpolarization	0.1–20 µM	[19]
			46.3 µM/1–1000 μM	[14]
			not specified	[51]
**Hesperidin**	Rat ileum/cecum (in vivo and ex vivo)	Contraction/MLCK activation, MLC20-P activation, COX-2/iNOS inhibition	2.5–160 µM	[15]
			not specified	[52]
**Hesperetin**	Rat jejunum (ex vivo)	Relaxation/K_ATP_ and NO pathway, prostaglandin modulation	10–100 µM	[53]
**Quercetin**	Guinea pig intestine, Mouse stomach, Human gastric strips (ex vivo); Rat colon and ileum (ex vivo)	Relaxation/Ca^2+^ channel antagonism, NO/opioid signaling; directly through K_ATP_ channels	10–300 µM	[19]
			4.3 µM/0.1–100 µM	[9]
			150–300 µM	[12]
			5.4 µM/0.1–100 µM	[13]
**Kaempferol**	Rat colon, jejunum, trachea, bladder (in silico, in vivo, ex vivo)	Relaxation/Ca^2+^ channel antagonism, K_V_ channels	not specified	[11]
			150–300 µM	[12]
			not specified	[54]
**Myricetin**	Rat colon and ileum (ex vivo)	Relaxation/Ca^2+^ channel blockade K_ATP_ channel activation	150–300 µM	[12]
			not specified	[11]
**Isorhamnetin**	Rat jejunum, bladder, rat ileum (in silico, ex vivo, and in vivo)	Relaxation/Ca^2+^ antagonism	not specified	[55]
**Catechin**	Rabbit jejunum, Mouse stomach; Rat fundus (ex vivo); Humans (in vivo)	Relaxation/Ca^2+^ channel blockade, NO generation	7.39 mM/1–30 mM	[16]
			13 µM/0.1–100 µM	[9]
			20 µM	[56]
**Epicatechin**	Rat jejunum (ex vivo, in vivo)	Relaxation/Ca^2+^ channel antagonism, MLCK binding	not specified	[17]
**Daidzein**	Rat jejunum; Guinea pig stomach (ex vivo)	Relaxation/Ca^2+^ channel antagonism, adrenergic signaling	1–300 µM	[57]
			5–160 μM	[18]
**Genistein**	Guinea pig intestine, Mouse stomach (ex vivo)	Relaxation/Excitation-contraction uncoupling	10–300 µM	[19]
			1.5 µM/0.1–100 µM	[9]
**Formononetin**	Rat aorta (ex vivo)	Relaxation/Endothelium/NO-dependent mechanism and -independent via BK, K_ATP_ activation	0.1–100 μM	[58]
**Pelargonidin**	Endothelial cells (ex vivo)	Relaxation/NO release from the endothelium	not specified	[59]
**Cyanidin-3-*O*-rutinoside**	Rat ileum (ex vivo)	Relaxation/VGCC blockade or interference with intracellular Ca^2+^ mobilization	3.23 mg/mL 0.01–3 mg/mL	[60]
**Resveratrol**	Rat uterus; Rat interstinal artery; Human gastric strips (ex vivo)	Relaxation/BK activation; L-type Ca^2+^ inhibition	0.01–50 μM	[26]
			not specified	[61]
			178 μM/0.1–100 µM	[25]
**Dibenzocyclooctadiene lignans**	Guinea pig ileum (ex vivo)	Relaxation/muscarinic receptors, intracellular Ca^2+^ mobilization and L-type Ca^2+^ inhibition	EC_20_ = 2.2 μM EC_50_ = 6.6 μM	[62]
**Arctigenin**	Rat ileum (ex vivo)	Relaxation/L-type Ca^2+^ inhibition	1–40 μM	[63]
**Trachelogenin**	Rat ileum (ex vivo)	Relaxation/L-type Ca^2+^ inhibition	0.5–20 μM	[63]
**Caffeic acid**	Rat aorta, uterus, and ileum (ex vivo)	Relaxation/Serotonergic, muscarinic receptors; L-type Ca^2+^ channels inhibition	2.1 mM/0.03–7 mM	[23]
**Rosmarinic acid**	Mouse colon (in vivo)	Relaxation/Downregulation of MLCK, ROCK	not specified	[24]

**Table 2:** Effects of polyphenol-rich plant extracts on gastrointestinal smooth muscle contractility.

Extract/Plant	Tissue/Model	Main Effect/Mechanism	EC_50_ and/or Reported Concentration	Reference
***Zingiber officinale* (Ginger)**	Mouse ileum, colon, LES; Rat colon (ex vivo); Humans, mouse (in vivo)	Relaxation/LES Contraction M3 and 5-HT_3_ receptor non-competitive antagonism L-type Ca^2+^ channel inhibition	0.001–1000 µM	[64]
			2.7 µM/1–30 µM	[65]
			not specified	[66]
			25–100 µM	[67]
			not specified	[68]
			3–30 µM	[69]
			not specified	[70]
***Curcuma longa* (Tumeric)**	Mouse ileum and colon, pulmonary artery, and ileum; Rat uterus (ex vivo)	Relaxation/Ca^2+^ channel blockade; non-competitive antagonism of cholinergic, histaminergic, and serotonergic receptors	0.021; 0.089 µM	[71]
			0.03; 0.04 mg/mL	[72]
			0.110 mg/mL/0.03–0.3 mg/mL	[73]
			12.9 µM	[74]
			not specified	[75]
***Bidens tripartita***	Porcine jejunum (ex vivo)	Contraction/Enhanced ACh response;	0.001–100 μM	[76]
***Roman Chamomile***	Guinea pig ileum; rat gut; human gut (ex vivo)	Relaxation/Direct smooth muscle relaxation	2–200 µg/mL	[77]
***Catha edulis***	Rat colon and ileum (ex vivo)	Relaxation/Ca^2+^ channel antagonism	0.05–0.5 mg/mL	[12]
***Tamarix dioica***	Rat and rabbit jejunum, trachea, aorta (ex vivo)	Relaxation/K_ATP_ channel activation; Ca^2+^ channel antagonism	0.334 mg/mL/0.3–3.0 mg/mL	[11]
***Citrullus lanatus***	Rabbit jejunum (in silico, ex vivo, in vivo)	Relaxation/Ca^2+^ channel antagonism	0.02482 mg/mL	[54]
***Cucumis melo***	Rabbit jejunum, trachea (in silico, ex vivo, in vivo)	Relaxation/Ca^2+^ antagonism, MAPK/PI3K targets	0.3–3 mg/mL	[78]
***Achillea millefolium***	Guinea pig ileum (ex vivo)	Relaxation/Ca^2+^ channel antagonism (quercetin, apigenin)	4.5 μM	[50]
***Baccharis conferta***	Guinea pig ileum (ex vivo)	Relaxation/histamine-dependent	123 and 234 μM	[49]
***Berberis lycium***	Rabbit jejunum, bladder, rat ileum (in silico, ex vivo, in vivo)	Relaxation/Ca^2+^ antagonism	0.3–3 mg/mL	[55]
***Melissa officinalis***	Rat ileum (ex vivo), chicken gut	Contraction/Relaxation/Potentiation of ACh-induced contraction	19 ng/mL/2.5–75 ng/mL	[79]
			0.1–100 µg/mL	[80]
***Salvia sclarea***	Rat ileum, trachea (in silico, ex vivo)	Relaxation/Ca^2+^ channel antagonism	2.4–5.8 mg/mL/0.005–1.5 mg/mL	[81]

### 5.1. Flavones: Potent Modulators of GI Smooth Muscle Activity

#### 5.1.1. Luteolin

Luteolin, a prevalent flavone abundant in numerous herbs and vegetables, has demonstrated a significant inhibitory impact on GI smooth muscle contractility. In murine models, luteolin dose-dependently attenuated both colonic smooth muscle motility and enteric neural activity, specifically influencing colonic motor complexes. The central mechanism underpinning this effect involves the inhibition of L-type calcium channels, as evidenced by its partial reversal with the known channel agonist, BayK8644. Crucially, luteolin’s relaxant activity remained insensitive to pharmacological blockade of various potassium channels (TEA, apamin, glibenclamide), voltage-gated sodium channels (TTX), NO synthase (L-NAME), NO-sensitive guanylyl cyclase (ODQ), and ANO1 channels [10].

#### 5.1.2. Apigenin

Apigenin, a prominent flavone widely present in botanical sources such as chamomile, parsley, and celery, demonstrates a broad range of myorelaxant effects throughout the GI tract. Ex vivo investigations utilizing isolated gut tissues have established apigenin as one of the most potent flavonoids in promoting smooth muscle relaxation, notably surpassing genistein, quercetin, and naringenin in efficacy. Its relaxant action largely proceeds independently of classical neural or systemic biochemical pathways, as evidenced by its persistence even in the presence of inhibitors of sodium channels (tetrodotoxin, TTX), NO synthase (L-NAME), cyclooxygenase (indomethacin), and various potassium channels (TEA). Structural elucidation has underscored the critical contribution of the C2=C3 double bond and specific hydroxyl substitutions to apigenin’s exceptional activity, aligning well with broader structure-activity relationship patterns identified within the flavonoid class [9].

Furthermore, apigenin has been repeatedly isolated and identified as a major active principle within numerous plant extracts celebrated for their antispasmodic properties. For instance, studies on *Achillea millefolium* and *Baccharis conferta* have successfully isolated apigenin and its derivatives from fractions displaying pronounced inhibitory effects on smooth muscle contraction. In the case of *B. conferta*, apigenin significantly contributed to the extract’s overall dose-dependent spasmolytic activity [49,50].

### 5.2. Flavanones: Diverse Influences on GI Motility

#### 5.2.1. Naringenin

Naringenin is a citrus-derived flavanone that consistently exerts a concentration-dependent relaxant effect on GI smooth muscle. In isolated rat colon preparations, naringenin effectively inhibited spontaneous contractions and suppressed calcium-induced contractile responses at a concentration of 100 µM. Patch-clamp electrophysiology confirmed that this relaxant effect critically involves the activation of BK channels, as its effect was abolished by both the non-selective potassium channel blocker TEA and the specific BK channel inhibitor iberiotoxin. Furthermore, naringenin was observed to induce hyperpolarization of colonic smooth muscle cells, providing additional evidence for its role in modulating membrane potential [14].

In vivo investigations have further proved naringenin’s influence, demonstrating its efficacy in slowing neostigmine-enhanced colonic transit in rats. This finding highlights its promising therapeutic potential in conditions characterized by GI hypermotility. Additionally, plant extracts such as those from *Varronia dardani*, where naringenin is a primary constituent, have exhibited non-selective spasmolytic activity in the rat uterus, suggesting that naringenin significantly contributes to the broader antispasmodic effects of various flavonoid-rich botanicals [43].

Intriguingly, when comparatively assessed with other flavonoids in guinea pig intestinal peristalsis models, naringenin elicited a moderate inhibition of distension sensitivity, yet demonstrated less pronounced effects on peristaltic propulsion than apigenin or genistein [19].

#### 5.2.2. Hesperidin

Hesperidin has been extensively investigated for its capacity to improve GI motility via diverse mechanisms. In a rat model of postoperative ileus, hesperidin significantly enhanced both gastric emptying and intestinal transit. Concurrently, in isolated ileum and cecum tissues, it increased the amplitude, but notably not the frequency, of spontaneous contractions. These pro-contractile effects were effectively antagonized by the MLCK inhibitor ML-7 and the Ca^2+^ channel blocker verapamil, unequivocally indicating mediation via intracellular calcium signaling and MLCK activation. Furthermore, hesperidin was observed to augment MLC phosphorylation and simultaneously attenuate the expression of inflammatory markers, including iNOS and COX-2, within the gut wall [15].

Complementary evidence from a loperamide-induced constipation model revealed that hesperidin improved stool consistency and accelerated colonic transit without influencing food intake. Mechanistically, hesperidin upregulated the expression of 5-HT_4_ receptors and downstream cAMP/PKA/CREB signaling cascade in smooth muscle cells, thereby contributing to both enhanced motility and reduced inflammation [52].

#### 5.2.3. Hesperetin

Hesperetin, the aglycone of hesperidin, demonstrates potent antispasmodic properties in isolated rat mesenteric jejunum tissues. It effectively inhibited both ACh- and KCl-induced contractions in a dose-dependent and reversible manner. The spasmolytic activity of hesperetin was significantly attenuated by 4-aminopyridine (4-AP), glibenclamide, and L-NAME, thereby implicating VGCCs and K_ATP_ channels, and the NO signaling pathway in its mechanism of action. Intriguingly, its activity remained unaffected by inhibitors such as apamin and TEA, suggesting a selective rather than broad action on potassium channels. Furthermore, indomethacin augmented hesperetin’s relaxant effect, indicating a potential interplay with prostaglandin pathways [53].

### 5.3. Flavonols: Prevalent Modulators of GI Motility

#### 5.3.1. Quercetin

Quercetin, arguably one of the most thoroughly investigated flavonols, exerts modulatory effects on GI motility, with compelling evidence indicating both direct smooth muscle relaxation and significant enteric neural involvement. In guinea pig intestinal models, quercetin dose-dependently inhibited peristaltic activity. This effect was primarily characterized by a notable reduction in distension sensitivity, while having a limited impact on active peristaltic propulsion. The observed antiperistaltic effect was partially reversed by apamin, L-NAME, and naloxone (an opioid receptor antagonist), collectively suggesting the involvement of SK channels, endogenous NO synthesis, and opioid receptor pathways [19].

In mouse stomach models, quercetin induced a reversible, dose-dependent relaxation of smooth muscle. Structural comparisons have elucidated that its efficacy diminishes upon glycosylation or saturation of the C2-C3 double bond, underscoring the critical role of specific structural motifs in its bioactivity. In vitro investigations utilizing *A. millefolium* extracts further confirmed quercetin’s potent spasmolytic effect (EC_50_ ≈ 7.8 μM), with the underlying mechanism strongly implicating Ca^2+^ channel blockade [9].

Notably, quercetin has been consistently identified as a major bioactive constituent in numerous polyphenol-rich extracts, including those from *Catha edulis*, *Citrullus lanatus*, and *Cucumis melo*, all of which demonstrated significant inhibitory effects on smooth muscle contractility. These effects frequently mimicked the pharmacological profile of established Ca^2+^ channel blockers, such as verapamil [12].

A recent study in our lab sought to clarify the mechanistic basis of quercetin’s relaxant effects, utilizing a more clinically relevant model of human gastric smooth muscle tissue. Our research was the first to definitively establish that quercetin-induced relaxation of human gastric smooth muscle is mediated directly through K_ATP_ channels. We demonstrated this using glibenclamide, a specific K_ATP_ channel blocker, which significantly inhibited the relaxant effect. We provided crucial evidence that this relaxing effect is independent of the NO pathway, as neither NOS nor guanylyl cyclase blockers had any inhibitory effect. This distinguishes quercetin’s mechanism from many other vasodilators and GI relaxants. We identified that other potassium channels, specifically BK and SK channels, also modulate quercetin’s effects, as their blockade extended the relaxation response. Tamoxifen also increased muscle relaxation, suggesting a role for estrogen-related receptors. We connected our findings to a direct clinical application, positioning quercetin as a promising nutraceutical for the treatment of functional dyspepsia and other gastric motility disturbances. This provides a clear, translational link from our basic science research to potential therapeutic use [13].

#### 5.3.2. Kaempferol

Kaempferol, another ubiquitously distributed flavonol, has consistently demonstrated robust smooth muscle relaxant properties across diverse in vitro and in vivo experimental models. It has been identified as a key active compound within extracts from *Tamarix dioica*, *C. lanatus*, and *C. melo*, significantly contributing to their observed spasmolytic effects. These actions predominantly appear to be mediated through direct potassium channel antagonism. Specifically, these extracts consistently inhibited high K^+^-induced contractions and induced rightward shifts in calcium dose–response curves in isolated jejunum, trachea, and bladder tissues, strongly indicating a shared underlying mechanism involving VGCCs [11].

Advanced investigations, including gene network analyses and molecular docking studies, have further implicated kaempferol in modulating the pathways crucial for intracellular calcium homeostasis and inflammation. Its consistent presence in polyphenol-rich botanical mixtures, which are associated with antidiarrheal, antiperistaltic, and antisecretory properties, underscores its significant therapeutic potential in the clinical management of functional GI disorders. Furthermore, within *C. edulis* extracts, kaempferol was definitively confirmed via NMR spectroscopy as one of the principal active constituents responsible for blocking calcium-dependent contractions in rat intestine, once again mirroring the established pharmacological action of verapamil [17].

#### 5.3.3. Myricetin

Myricetin, while less comprehensively studied in isolation compared to other flavonols, was consistently identified as a prominent bioactive constituent, alongside quercetin and kaempferol, in *C. edulis* extracts. This extract notably reduced spontaneous contractions in the isolated rat colon and ileum. The extract’s effect remained unaltered by TTX, a sodium channel blocker, thereby suggesting a direct action on smooth muscle cells rather than neurogenic mediation. Analogous to its structural counterparts, myricetin is posited to exert its effects through Ca^2+^ channel inhibition, although detailed specific mechanistic data remain scarce [12].

Myricetin was also detected in *T. dioica* extracts, where its involvement in K_ATP_ channel activation was hypothesized as a component of a broader spasmolytic mechanism. Nevertheless, due to the inherent complexity of such crude extracts, the precise individual contribution of myricetin to these observed effects warrants further study [11].

#### 5.3.4. Isorhamnetin

Isorhamnetin, a methylated metabolite of quercetin, has been identified within the polyphenolic fraction of *Berberis lycium*. Comprehensive investigations, spanning in silico predictions, in vitro assays, and in vivo models, have demonstrated that this flavonol significantly contributes to the extract’s overall antispasmodic and antidiarrheal effects. Experimental data specifically revealed its capacity to inhibit jejunal and bladder contractions, exert a dual blocking mechanism of Ca^2+^ channels, and muscarinic signaling pathways. Furthermore, the extract’s ability to downregulate pro-inflammatory markers such as IL-1β and TNF-α collectively reinforces the substantial therapeutic potential of isorhamnetin-containing preparations for managing various GI motility disorders [55].

### 5.4. Flavanols: Key Calcium Antagonists in GI Smooth Muscle

#### 5.4.1. Catechin

Catechin, a principal dietary flavanol found in tea, cocoa, and various fruits, consistently induced smooth muscle relaxation across a range of GI models. In isolated rabbit jejunum, catechin dose-dependently inhibited both spontaneous and high K^+^-induced contractions, with a preferential effect against the latter. This was reflected in a rightward shift in the calcium concentration–response curve, closely mimicking the action of verapamil, a classical Ca^2+^ channel blocker. These effects were reproduced in other smooth muscle tissues, including rat stomach fundus and guinea pig ileum, supporting catechin’s consistent myorelaxant action via Ca^2+^ antagonist activity [16].

Mechanistic studies indicated that catechin’s spasmolytic effect is not mediated by neural pathways or classic relaxant mediators. Its activity was unaffected by TTX, L-NAME (NO synthase inhibitor), indomethacin (COX inhibitor), or tetraethylammonium (non-selective K^+^ channel blocker), suggesting a direct action on smooth muscle calcium dynamics [9].

Additionally, studies simulating gastric conditions demonstrated that catechin, in the presence of nitrite, facilitates NO production, leading to gastric muscle relaxation [56].

#### 5.4.2. Epicatechin

Epicatechin shares structural similarity with catechin and demonstrates comparable spasmolytic properties. In studies using *C. lanatus* seed extracts, which contain epicatechin among other flavonoids, a dose-dependent relaxation of K^+^-induced contractions was observed in isolated jejunum and tracheal tissues. Like catechin, epicatechin shifted calcium dose–response curves and suppressed calcium-dependent contractions, indicating Ca^2+^ channel antagonism.

Moreover, in silico analyses revealed strong binding affinity of epicatechin to several calcium-regulatory targets, including VGCCs and MLCK. These interactions provide a molecular basis for its relaxant effects. The extract also demonstrated in vivo efficacy by reducing intestinal motility and fluid secretion, consistent with the observed in vitro inhibition of smooth muscle activity [17].

### 5.5. Isoflavones—Unique Structures with Diverse Pharmacological Profiles

#### 5.5.1. Daidzein

Daidzein, a prominent isoflavone sourced from soy, consistently inhibits intestinal motility in both physiological and hypercontractile states. In isolated jejunal smooth muscle preparations, daidzein reduced spontaneous contractility and suppressed contractions induced by various agonists, including ACh, histamine, erythromycin, and high extracellular Ca^2+^ concentrations. The relaxant effect was dependent on extracellular calcium, as it was abolished in Ca^2+^-free media and its pharmacological profile closely mimicked that of the L-type Ca^2+^ channel blocker, verapamil. Furthermore, a role for adrenergic receptors was indicated, as the effect was partially attenuated by both the α-adrenergic antagonist phentolamine and the β-adrenergic antagonist propranolol [18].

Complementary patch-clamp studies on guinea pig gastric myocytes revealed that daidzein dose-dependently suppresses voltage-dependent Ba^2+^ currents. This finding supports the hypothesis that daidzein’s action involves tyrosine kinase inhibition, which can, in turn, reduce calcium influx via L-type Ca^2+^ channels. Importantly, daidzein’s effect remained unaltered by inhibition of nitric oxide synthase, underscoring that its primary mechanism is largely independent of nitric oxide-mediated signaling pathways [57].

#### 5.5.2. Genistein

Genistein, a structural analog of daidzein, has been shown to exert potent antiperistaltic effects in isolated guinea pig intestine. In comparative analyses with other flavonoids, genistein displayed a unique pharmacological profile, characterized by the inhibition of both distension sensitivity and peristaltic propulsion, which is a pattern shared exclusively with apigenin. In contrast to the effects of quercetin, genistein’s actions were not attenuated by apamin, naloxone, or L-NAME, thereby suggesting a minimal role for neural inhibitory pathways or nitric oxide signaling [19].

The observation that neostigmine successfully restored genistein-inhibited peristalsis, yet failed to reverse the effects of quercetin, underscores a distinct mechanistic difference likely localized to the excitation-contraction coupling process within the smooth muscle cell itself. Furthermore, studies on the gastric muscle have revealed that genistein produces a strong relaxant effect that is independent of neural blockade, providing additional evidence for its direct action on smooth muscle cells [9].

#### 5.5.3. Formononetin

Formononetin, a naturally occurring isoflavone, induces vascular smooth muscle relaxation through a dual mechanism involving both endothelium-dependent and -independent pathways. Although the bulk of the evidence is derived from aortic tissue, its relevance to GI smooth muscle is underscored by the shared molecular targets, particularly K_ATP_ and BK channels.

In rat aortic rings, formononetin-induced vasodilation was attenuated by L-NAME and methylene blue, thereby implicating endothelial nitric oxide synthase (eNOS) and the NO-cGMP signaling pathway. In contrast, in endothelium-denuded tissues, the relaxant effect persisted, but was abrogated by K_ATP_ channel blocker glibenclamide and the specific BK channel inhibitor iberiotoxin. Subsequent cellular assays definitively confirmed that formononetin directly activates these potassium channels in smooth muscle cells [58].

### 5.6. Anthocyanins—Limited Direct Contractility Data

Although anthocyanin-rich foods and extracts have been extensively studied for their anti-inflammatory, antioxidant, and microbiota-modulating effects in the GI tract [82], direct experimental evidence of their actions on isolated smooth muscle contractility remains scarce. Their poor intestinal absorption and extensive metabolism further complicate the interpretation of any observed motility effects in vivo, as many biological actions may arise from microbial degradation products rather than the parent compounds.

#### 5.6.1. Pelargonidin

Pelargonidin, a red-pigmented anthocyanin found in foods such as strawberries and red radishes, has been implicated in the modulation of vascular tone through NO-dependent pathways. While direct evidence of its effect on gastrointestinal smooth muscle remains sparse, valuable insights can be extrapolated from studies on vascular endothelial cells.

In an investigation of red wine polyphenols, pelargonidin was identified as a potent stimulator of endothelial NO production. Using electron paramagnetic resonance spectroscopy, researchers confirmed that pelargonidin promoted a measurable intracellular calcium influx, which in turn increased NO synthesis in endothelial cells, a response that was blocked by inhibition of NO synthase.

Crucially, this pro-NO effect was not observed in isolated smooth muscle cells, which strongly suggests that pelargonidin’s primary mechanism involves an endothelium-mediated signaling cascade [59].

Although these findings do not provide direct confirmation of an effect on GI smooth muscle, they compellingly suggest that pelargonidin may contribute to smooth muscle relaxation through an endothelium-derived NO signaling pathway. This mechanism could be particularly relevant in the highly vascularized regions of the GI tract. Further, targeted investigations are warranted to determine whether a similar mechanism is operational in the regulation of enteric smooth muscle.

#### 5.6.2. Cyanidin-3-*O*-Rutinoside

The clearest ex vivo contractility evidence derives from studies using anthocyanin-rich extract preparations rather than purified molecules. In an experiment employing isolated rat ileum, Miladinovic et al. (2018) [60] demonstrated that blackcurrant (*Ribes nigrum* L.) juice, containing cyanidin-3-*O*-rutinoside and delphinidin-3-*O*-rutinoside as dominant pigments, produced a concentration-dependent inhibition of BaCl_2_- and KCl-induced contractions. The extract also relaxed spontaneous phasic activity and reduced ACh-induced tone, suggesting that its spasmolytic action might involve VGCC blockade or interference with intracellular Ca^2+^ mobilization [60]. However, the complex composition of the juice—rich in other polyphenols, organic acids, and sugars—precludes attribution of the relaxant effect exclusively to anthocyanins.

In the case of anthocyanins, specific contractility data are sparse. Most of the anthocyanin literature focuses on anti-inflammatory, antioxidant, and barrier effects rather than direct experimental studies of smooth muscle contraction in the GI tract [83]. These gaps suggest that more targeted studies are needed to clarify whether anthocyanins exert consistent spasmolytic or pro-motility effects in isolated GI smooth muscle and in vivo models.

## 6. Effects of Stilbenes: Resveratrol as a Multi-Targeted Spasmolytic Agent

Resveratrol, a key representative of stilbene polyphenol, found in red wine and various plant sources, is studied for its wide-ranging cardiovascular, anti-inflammatory, and antioxidant properties. Within the context of GI physiology, resveratrol demonstrates potent spasmolytic effects on smooth muscle tissue, mediated by distinct mechanisms that vary depending on the experimental model.

In rat models, resveratrol additionally decreased basal tone and reduced the contractile amplitude of GI smooth muscle. These effects were partially reversed by antagonists of α-adrenergic receptors (phentolamine), NO synthase inhibitors, and K_ATP_ channel blockers (glibenclamide), which suggests a broader mechanistic profile involving L-type Ca^2+^ channels and cAMP signaling pathways. Consistent with these findings, a rightward shift in calcium dose–response curves provided further confirmation of its inhibitory effect on calcium influx [26].

Furthermore, in models of intestinal ischemia/reperfusion injury, resveratrol successfully restored impaired contractility. This effect was coupled with a reduction in oxidative stress and pro-inflammatory cytokines (IL-1β, TNF, MPO), while concurrently replenishing glutathione levels [61].

To clarify the mechanistic basis of resveratrol’s relaxant effects, our study utilized a clinically relevant human gastric smooth muscle model. We were the first to investigate the direct relaxant effects of resveratrol. This provides crucial translational data and a more clinically relevant perspective compared to studies using animal models. We established that resveratrol’s relaxing effects are primarily mediated by the activation of BK channels. This was confirmed by the fact that BK channel blockers like TEA, iberiotoxin, and charybdotoxin significantly inhibited the relaxation response. We ruled out the involvement of other common relaxant pathways, including the NO signaling cascade and other potassium channels (K_ATP_ and K_V_), by showing that their respective blockers (L-NAME, L-NNA, ODQ, glibenclamide, and 4-AP) did not affect resveratrol’s action. The relaxant effect was also independent of tamoxifen-sensitive receptors [25].

## 7. Effects of Lignans: Multi-Pathway Modulation of Smooth Muscle Contractility

In the GI system, emerging evidence suggests that lignans can directly influence smooth muscle contractility through multiple pathways.

### 7.1. Dibenzocyclooctadiene Lignans

*Schisandra chinensis*, a medicinal plant rich in dibenzocyclooctadiene lignans, has been investigated for its effects on intestinal motility. Yang et al. (2011) [62] demonstrated that crude extracts of *S. chinensis* and its major lignans, including schisandrin, significantly relaxed agonist-induced contractions in isolated guinea pig ileum. Specifically, schisandrin produced a relaxant effect on 5-HT-induced contractions with an EC_50_ value of 40.7 ± 5.4 mM. The inhibitory actions against contractions triggered by acetylcholine, histamine, and KCl suggest a mechanism involving both receptor-mediated and direct smooth muscle pathways. The study proposed that these lignans exert their relaxant effects by modulating calcium influx across the smooth muscle membrane, consistent with Ca^2+^ channel-blocking activity.

### 7.2. Dibenzylbutyrolactone Lignans

More recently, Koech et al. (2022) [63] evaluated the dibenzylbutyrolactone lignans arctigenin and trachelogenin in isolated rat ileum preparations. Both compounds decreased the frequency of spontaneous contractions in a concentration-dependent manner, prolonging the time between them. Their effects were comparable to the L-type calcium channel blocker verapamil, further supporting a role for calcium influx inhibition. Interestingly, the primary effect on contraction frequency, with a lesser effect on amplitude, indicates that these lignans principally alter the pacemaker activity of the intestinal smooth muscle.

These findings from different structural types of lignans—dibenzocyclooctadiene [62] and dibenzylbutyrolactone [63]—underscore their potential as smooth muscle relaxants in the GI tract. Their mechanisms appear to converge on calcium channel modulation, though contributions from receptor-level interactions cannot be excluded. Despite these promising results, the available data are limited, and further comparative studies are warranted to establish the pharmacological relevance of lignans in managing GI motility disorders.

## 8. Effects of Phenolic Acids: Diverse Mechanisms of Motility Modulation

### 8.1. Hydroxycinnamic Acids: Modulators of Contractility and Inflammation

#### 8.1.1. Caffeic Acid

Caffeic acid, a hydroxycinnamic acid derivative widely present in coffee, pears, and apples, demonstrates significant relaxant activity on smooth muscle across multiple organ systems. In an organ bath study comparing the aorta, uterus, and ileum, caffeic acid induced dose-dependent relaxation in preparations precontracted with diverse agonists, including high K^+^ concentration, phenylephrine, oxytocin, and carbachol. Notably, the ileum exhibited the highest sensitivity to caffeic acid, with an EC_50_ of approximately 2.0 mM. These results suggest that caffeic acid exerts complex antispasmodic effects with various mechanisms involved [23].

#### 8.1.2. Rosmarinic Acid

Rosmarinic acid, a major phenolic constituent of *Salvia sclarea* and *Melissa officinalis*, has demonstrated promising therapeutic effects in models of inflammatory bowel disease. In vivo studies have shown that it effectively attenuates intestinal inflammation, restores epithelial barrier function, and modulates the composition of the gut microbiota. Molecular analyses confirmed that rosmarinic acid downregulates the expression of key contractility-associated genes, including ROCK, MLCK, as well as major inflammatory mediators (TNF-α, IL-1β, and IL-6) [24].

## 9. Effects of Polyphenol-Rich Extracts and Mixed Compositions: Diverse Actions on GI Motility

Polyphenol-rich botanical extracts and complex natural compositions are widely used for their medicinal properties, including their impact on GI motility. These extracts often contain a synergistic blend of compounds, such as flavonoids, gingerols, and curcuminoids, which exert multifaceted effects on GI smooth muscle. Their mechanisms range from direct modulation of ion channels and receptor antagonism to indirect anti-inflammatory actions (Table 2). This section explores the effects of two prominent plant extracts and the active compounds that contribute to their complex pharmacological profiles.

### 9.1. Zingiber officinale (Ginger) Extract

Gingerols, particularly 6-gingerol, are identified as the primary active compounds in *Zingiber officinale* (ginger) extract, which is traditionally used to alleviate nausea and GI discomfort. Studies on mouse ileum and colon have shown that the extract, through the action of its gingerol and shogaol components, potently inhibits smooth muscle contractions via a non-competitive antagonism of muscarinic M3 and serotonin 5-HT_3_ receptors. Furthermore, the extract has been observed to increase tone in the lower esophageal sphincter (LES), suggesting its potential as a therapeutic agent for gastroesophageal reflux disease (GERD) [69].

Patch-clamp studies in rat colon have provided a molecular basis for these effects, demonstrating that gingerol directly inhibits L-type Ca^2+^ channel currents in smooth muscle cells. This action was attenuated by the Ca^2+^ channel blocker nifedipine, confirming a direct Ca^2^-channel-related mechanism. The compound was found to shift the voltage-dependence of channel activation, further supporting its role as a direct modulator of these ion channels [67].

Complementary functional studies on 5-HT_3_ receptor binding and function have shown that gingerols and shogaols inhibit serotonin-induced cation flux and ileal contraction. This is likely via interaction with modulatory rather than orthosteric binding sites on the receptor complex [64,65].

Evidence from clinical trials demonstrates that ginger extract significantly accelerates gastric emptying and stimulates antral contractions. This prokinetic effect, particularly relevant to conditions like nausea and vomiting in pregnancy, positions ginger as a harmless and effective alternative therapy for managing these symptoms [84,85,86]. These clinical outcomes are consistent with ginger’s known mechanisms, including the targeting of signaling molecules and receptors that regulate GI smooth muscle activity [68,70].

### 9.2. Curcuma longa (Turmeric) Extract

Curcuminoids, the primary active polyphenolic pigments extracted from *Curcuma longa* (turmeric), are responsible for its strong antispasmodic activity, observed in both guinea pig ileum and rat uterus preparations. The extract, rich in these compounds, dose-dependently induces marked relaxation of GI smooth muscle in mice, acting through non-competitive antagonism of cholinergic (carbachol), histaminergic, and serotonergic pathways. Curcuminoids also inhibit K^+^-induced contractions by blocking L-type Ca^2+^ channels, producing potent spasmolysis in mouse ileum and colon [62]. In a murine colitis model, Curcuma extract suppressed both spontaneous and carbachol-induced colonic contractions. These reversible effects in inflamed tissue were accompanied by a partial recovery of contractile activity independent of the extract’s anti-inflammatory properties [72].

Pharmacological evaluations in isolated rabbit jejunum and other smooth muscle tissues [73] showed that turmeric extract and curcumin inhibited both spontaneous and depolarization-induced contractions, a pattern consistent with calcium-channel blockade.

Structure–activity studies in guinea-pig ileum confirmed that bisdemethoxycurcumin is the most potent spasmolytic, followed by curcumin and demethoxycurcumin, whereas tetrahydrocurcumin and hydrolysis products were largely inactive [74].

Review articles further summarize curcumin’s capacity to reduce hypercontractility, normalize disordered motility, and protect neuromuscular transmission in inflammatory states. These effects likely result from both direct smooth-muscle relaxation and indirect modulation of oxidative and inflammatory processes, as noted across clinical and experimental reports [73,75].

### 9.3. Bidens tripartita

Extracts of *Bidens tripartita*, a plant traditionally used for digestive ailments, have demonstrated potent prokinetic effects in ex vivo models. From the plant’s aerial parts, researchers isolated several flavonoids, including luteolin, cymaroside, and flavanomarein, and prepared six different extracts and fractions. The flavonoid-rich extracts enhanced both spontaneous and ACh-induced contractions of porcine isolated jejunal smooth muscle. Notably, individual flavonoid constituents, particularly cymaroside, amplified the ACh response by up to 250%, suggesting a substantial synergistic or potentiating effect [76]. Taken together, these findings indicate its potential as a prokinetic agent for managing hypomotility conditions such as constipation-dominant IBS or functional dyspepsia.

### 9.4. Roman Chamomile (Chamaemelum nobile)

Roman chamomile, a low perennial plant with a history of use in traditional medicine since the Middle Ages, is valued for its ability to treat spasms of the GI system. The limited experimental data available support its relaxant properties, with a single study demonstrating biphasic effects on GI smooth muscle in isolated organ bath experiments using guinea pig, rat, and human preparations [77].

However, the plant extract’s primary effect was a sustained relaxant action on precontracted smooth muscle. This effect was found to be flavonoid-dependent, and persisted even in the presence of β-adrenergic blockers. The relaxant properties of four flavonoids isolated from the plant material (hispidulin, luteolin, eupafolin, and apigenin) were observed across species, supporting the plant’s broad pharmacological relevance [77].

Further studies with chamomile essential oil showed only relaxant effects, indicating that its volatile constituents act through a mechanism distinct from the polyphenolic fraction.

### 9.5. Catha edulis

Crude extracts of *Catha edulis*, which are rich in flavonoids such as quercetin, kaempferol, and myricetin, significantly inhibited spontaneous contractions in rat colon and ileum. These effects were comparable to those induced by the calcium channel blocker verapamil and were unaffected by TTX, definitively indicating a direct action on smooth muscle rather than neural mediation. The extract reduced both K^+^-induced and Ca^2+^-stimulated contractions, suggesting that calcium channel blockade is the primary mechanism. The identity of the flavonoids responsible for this activity was confirmed by NMR spectroscopy [12].

### 9.6. Tamarix dioica

*Tamarix dioica* is traditionally used to manage various disorders related to smooth muscle in the GI, respiratory, and cardiovascular systems. It is useful in the treatment of diarrhea, dysentery, and inflammation. The methanolic extract of *T. dioica* has demonstrated a broad spectrum of pharmacological activities, including spasmolytic, bronchodilatory, vasorelaxant, and antidiarrheal effects. Phytochemical analysis revealed a high flavonoid content, with rutin, catechin, kaempferol, myricetin, and apigenin identified as principal constituents. Mechanistically, the extract’s relaxant effects appear to involve the activation of K_ATP_ channels, as its actions were inhibited by glibenclamide in ex vivo rat and rabbit jejunum models. In vivo experiments further validated its use in treating diarrheal syndromes, aligning with its ethnomedicinal application [11].

### 9.7. Citrullus lanatus (Watermelon Seeds)

Hydroethanolic extracts from watermelon seeds (*Citrullus lanatus*) are rich in polyphenolic compounds, including catechin, epicatechin, quercetin, and kaempferol. These extracts exhibit potent spasmolytic effects across various smooth muscle tissues. They effectively reduced K^+^-induced contractions and shifted calcium dose–response curves in rabbit isolated jejunum and tracheal preparations, providing clear evidence for calcium antagonism [54].

In vivo studies demonstrated that these extracts produce antiperistaltic, antidiarrheal, and antisecretory effects. Complementary in silico analyses have confirmed that these compounds target key calcium-related signaling proteins, thereby providing a molecular basis for their action as calcium channel antagonists [17].

### 9.8. Cucumis melo (Melon Seeds)

*Cucumis melo* is a nutritious and therapeutic fruit which has been used in traditional medicine for centuries in Pakistan, Iran, India, and China for GI, circulatory, neurological, and urogenital problems. A polyphenol-rich extract (rutin, kaempferol, quercetin, apigenin, and luteolin) derived from *C. melo* seeds demonstrated strong inhibitory effects on K^+^-induced smooth muscle contractions, a finding consistent with calcium antagonism. Gene network analyses identified high-affinity interactions between the flavonoids in the extract and genes associated with inflammatory signaling and calcium regulation [78].

### 9.9. Achillea millefolium (Yarrow)

Yarrow (*Achillea millefolium*) extracts inhibited contractions of guinea pig ileum in a dose-dependent manner, with quercetin, luteolin, and apigenin identified as the main active aglycones. The very low micromolar range of the EC_50_ values indicates their high potency. The primary mechanism of action is hypothesized to involve calcium channel blockade, with possible secondary modulation of inflammatory or receptor-mediated pathways [50].

### 9.10. Baccharis conferta

Ethanolic extracts of *Baccharis conferta*, particularly those enriched in flavonoids, exhibited potent spasmolytic activity in histamine-contracted intestinal tissues. The identified active constituents included apigenin, naringenin derivatives, and cirsimaritin [49].

### 9.11. Berberis lycium

*Berberis lycium* is a member of the Berberidaceae family and is highly esteemed in traditional remedies worldwide, with a long history of treating diarrhea and abdominal spasms. The flavonoid-rich hydromethanolic extract of *B. lycium* showed antispasmodic, antidiarrheal, and bronchodilatory activities in both in vitro and in vivo models. Network pharmacology studies linked its action to the modulation of inflammatory genes and calcium-regulating proteins. Functional assays confirmed both calcium channel blockade and the inhibition of carbachol-induced contractions. The extract’s comprehensive pharmacological profile suggests its potential utility in managing disorders involving both hypercontractile and inflammatory components [55].

### 9.12. Melissa officinalis (Lemon Balm)

*Melissa officinalis*, commonly known as lemon balm, is a traditional medicinal herb valued for its digestive, antispasmodic, and sedative properties. Its complex phytochemical profile includes mono-, sesqui-, and triterpenes, as well as a variety of phenolic compounds, with rosmarinic, chlorogenic, and lithospermic acids being the most abundant [70].

Rosmarinic acid has been linked to vasorelaxant effects in the isolated rat thoracic aorta, mediated primarily by NO-dependent mechanisms, with a possible involvement of prostacyclin and endothelium-derived hyperpolarizing factor (EDHF) pathways [87].

Recent investigations into intestinal smooth muscle have revealed a more complex, region-dependent action of *M. officinalis* extract. In isolated chicken proximal and distal jejunum, the crude extract generally enhanced ACh-induced contractility and increased spontaneous motor activity in the distal segment. Conversely, spontaneous contractions were reduced by as much as ~67% of control values in the proximal jejunum. Furthermore, testing individual phenolic acids (rosmarinic, chlorogenic, and lithospermic) showed predominantly myorelaxant effects on ACh-induced contractions. This contrasts with the extract’s overall myocontractile action, suggesting complex synergistic or antagonistic interactions among constituents that cannot be attributed to a single compound [80].

### 9.13. Salvia sclarea

*Salvia sclarea* (clary sage), traditionally valued for its antispasmodic and aromatic properties in managing digestive complaints, has also been applied in treating respiratory disorders and various inflammatory conditions. Phytochemical analyses of its methanolic extracts have identified several polyphenolic constituents, including rosmarinic and caffeic acids, apigenin, luteolin, and salvigenin, along with their respective glycosides, which likely contribute to its pharmacological profile [81].

Experimental studies on isolated rat ileum demonstrated that *S. sclarea* extracts reduced both spontaneous and induced contractions, including those triggered by potassium depolarization and acetylcholine. The observed inhibition of K^+^-induced contractions strongly suggests a mechanism involving the blockade of voltage-dependent L-type Ca^2+^ channels and the activation of K^+^ channels. Furthermore, the extracts relaxed tracheal smooth muscle, highlighting a notable bronchodilatory potential [81]. Molecular docking studies on these extracts implicated active components, such as flavonoid glycosides (e.g., apigenin-7-*O*-glucoside and luteolin-7-*O*-glucoside), as primary compounds responsible for targeting calcium channels.

## 10. Compounds Lacking Direct Experimental Data

Several polyphenolic compounds named in the manuscript are recognized for important biological activities in contexts outside the GI tract. Tangeretin has been studied for antioxidant, anti-inflammatory, and anticancer effects, including modulation of signalling pathways relevant to tumor growth and apoptosis [88]. Nobiletin and its metabolites show neuroprotective and cardiometabolic activity, with evidence for anti-inflammatory and cognitive benefits in preclinical models [89]. The isoflavone biochanin A exhibits phytoestrogenic, anti-inflammatory, and anticancer properties and has been proposed as a lead for metabolic and antiproliferative interventions [90]. Glycitein, a soy isoflavone, has demonstrable weak estrogenic activity and has been investigated in the context of endocrine modulation and bone health [91]. Among anthocyanidins, malvidin and its glycosides have been reported to possess antioxidant, anti-inflammatory, and phosphodiesterase-modulating activities relevant to cardiovascular and epithelial biology [92], while petunidin displays potent antioxidative and bone-protective effects in cellular and animal models of osteoclastogenesis and aging [93].

However, a critical gap exists in the current literature: no original experimental studies were found that specifically investigated their effects on GI smooth muscle contractility. Consequently, these compounds were excluded from detailed analysis within this review. This identified gap represents a significant opportunity for future research to explore their potential roles in modulating GI motility.

## 11. Classification of Experimental Models

For clarity within this review, the experimental models discussed and presented in Table 1 and Table 2 adhere to standard terminology:

In silico: Refers to computer-based simulations or computational analyses.

In vivo: Denotes experiments conducted in living organisms.

Ex vivo: Pertains to studies performed on tissues or organs freshly isolated from living animals and maintained under physiological conditions. While some authors may refer to such preparations as in vitro (in glass), this review adopts the term ex vivo to underscore the preservation of native tissue architecture and function, which is critical for assessing physiological relevance. The term in vitro refers to experiments conducted with isolated cells, subcellular components, or purified molecules in a controlled laboratory setting, like a test tube or Petri dish.

## 12. Clinical Implications and Limitations

Although extensive in vitro and ex vivo studies demonstrate the spasmolytic and contractility-modulating effects of polyphenols, translation to clinical practice remains limited. A major challenge is the bioavailability of polyphenols, which is often low due to poor intestinal absorption, extensive first-pass metabolism, and rapid elimination [7,48,94]. As a result, effective concentrations observed in preclinical models may not be achievable in vivo, and systemic exposure may differ substantially from that of isolated tissues.

Another important consideration is the potential for drug–polyphenol interactions. Polyphenols can influence drug-metabolizing enzymes and transporters, raising the possibility of altered pharmacokinetics when co-administered with conventional medications [95]. This is particularly relevant for GI and cardiovascular drugs, where overlapping mechanisms such as Ca^2+^ channel modulation or adrenergic signaling may result in synergistic or antagonistic effects.

Human data remain scarce, and most studies have evaluated polyphenol-rich extracts rather than isolated compounds. Extracts are chemically complex, and their effects depend on concentration, matrix composition, and possible synergism between constituents [7,48]. This complicates the attribution of activity to a specific molecule and limits the ability to draw firm mechanistic conclusions.

Despite a substantial body of mechanistic evidence, significant research gaps persist. While flavones and flavonols have been extensively studied, the effects of other important subclasses, such as anthocyanins and lignans, on GI smooth muscle remain largely unexamined [63]. Furthermore, the majority of findings are derived from in vitro or ex vivo experiments, with a scarcity of human studies [7,96]. The few human trials that exist often rely on complex extracts rather than isolated compounds, and a lack of standardized protocols and inconsistent reporting of concentrations further impede meaningful cross-study comparisons [7,96].

These factors contribute to key inconsistencies in the literature. Certain compounds demonstrate spasmolytic activity in one model, but excitatory effects in another, and extract-based results do not always align with data from pure compounds. These discrepancies underscore the complex interplay of synergistic effects and tissue-specific molecular targets [95,97].

These limitations underscore the need for well-designed clinical trials with standardized formulations, dose–response evaluations, and robust endpoints. Future research must also address pharmacokinetic–pharmacodynamic relationships and carefully monitor for drug–polyphenol interactions. Only then can the promising mechanistic insights from preclinical studies be successfully translated into clinically viable therapies [7,95,96].

## 13. Conclusions

The evidence reviewed here demonstrates that dietary polyphenols, especially flavonoids, modulate gastrointestinal smooth muscle contractility through various mechanisms. These effects range from prokinetic stimulation to potent spasmolytic activity, with the response often depending on chemical structure, concentration, and experimental context.

The most frequently implicated mechanistic targets include voltage-dependent L-type calcium channels, ATP-sensitive and large-conductance calcium-activated potassium channels, nitric oxide signaling, and cAMP/PKA- or MLCK-dependent phosphorylation cascades. Intriguingly, many polyphenols act directly on smooth muscle cells, bypassing neural input, while others modulate enteric neurotransmission or inflammatory pathways. The specific bioactivity of these compounds is significantly influenced by structural features, such as hydroxylation patterns, glycosylation, and the saturation of the C2-C3 double bond.

## 14. Future Directions

The traditional use of polyphenol-rich plant extracts for GI disorders is supported by evidence of their synergistic action. However, the complexity of these mixtures poses a challenge for precisely attributing their mechanisms. This underscores the need for future structure–activity studies and bioassay-guided fractionation to isolate and characterize individual contributions.

To advance the field, future studies should focus on standardized methodologies with quantitative evaluation of potency, comparative analyses across subclasses, and mechanistic exploration beyond Ca^2+^ channel antagonism, including receptor-level interactions and microbiota-derived metabolites. Above all, well-designed clinical trials are urgently needed to clarify translational potential, with attention to bioavailability, pharmacokinetics, and drug–polyphenol interactions.

This comprehensive review highlights that polyphenols are multi-target modulators of gastrointestinal motility, offering therapeutic potential for functional bowel disorders, postoperative ileus, and other related dysmotilities. Advancing this potential into clinical practice requires future studies to resolve key gaps in their in vivo bioactivity, site-specific pharmacokinetics, and potential interactions with conventional therapies.

## Data Availability

No new data were generated or analyzed in this study. Data sharing does not apply to this article as it is a review of previously published literature.

## Abbreviations

The following abbreviations are used in this manuscript:

4-AP	4-aminopyridine
5-HT	Serotonin receptors
AC	Adenylate cyclase
ACh	Acetylcholine
ADRA	Adrenergic receptors
BK	Large-conductance calcium-activated potassium channels
cGMP	Cyclic guanosine monophosphate
COX-2	Cyclooxygenase-2
CREB	cAMP response element-binding protein
DAG	diacylglycerol
EC_50_	half-maximal effective concentration
eNOS	Endothelial nitric oxide synthase
GI	Gastrointestinal
IBS	Irritable bowel syndrome
IK	Intermediate-conductance calcium-activated potassium channels
IL	Interleukin
iNOS	Inducible nitric oxide synthase
IP_3_	Inositol trisphosphate
IP_3_R	Inositol trisphosphate potassium receptor
K_ATP_	ATP-sensitive potassium channels
LES	Lower esophageal sphincter
L-NAME	N(G)-nitro-L-arginine methyl ester
M2, M3	Muscarinic acetylcholine receptors
MLC20	20 kDa regulatory light chains
MLCK	Myosin light chain kinase
MLCP	Myosin light chain phosphatase
MPO	Myeloperoxidase
NMR	Nuclear magnetic resonance spectroscopy
NO	Nitric oxide
NOS	Nitric oxide synthase
ODQ	NO-sensitive guanylyl cyclase
PIP_2_	phosphatidylinositol 4,5-bisphosphate
PKA	Protein kinase A
PKC	Protein kinase C
PKG	Protein kinase G
PLB	phospholamban
PLCβ	phospholipase C beta
ROCK	Rho-associated protein kinase
RyR	Ryanodine receptor
SERCA2a	sarco/endoplasmic reticulum Ca^2+^-ATPase 2a
sGC	Soluble guanylate cyclase
SK	Small-conductance calcium-activated potassium channels
SR	Sarcoplasmic reticulum
TEA	Triethylamine
TNF	Tumor necrosis factor
TTX	Tetrodotoxin
VGCC	Voltage-gated calcium channel
